# Camphor Oil Toxicity: A Case Report

**DOI:** 10.7759/cureus.47412

**Published:** 2023-10-21

**Authors:** Sarah G Jabbour, Nabiha A Mawlawi, Mehraj W Kuthbudeen, Salwa Y Aljanaahi

**Affiliations:** 1 Emergency Medicine, Rashid Hospital, Dubai, ARE

**Keywords:** airway protection, arrhythmias, wide complex qrs, generalized tonic clonic seizure, camphor oil

## Abstract

Camphor is a highly toxic ingredient that can be found in commonly used rubs and preparations such as Tiger balm and Vicks. There is a wide range of symptoms resulting from camphor oil toxicity, manifesting in sweating and agitation and can progress to more serious symptoms of seizures, cardiac arrhythmias, and cardiopulmonary arrest.

We present a 61-year-old male, who is a known case of major depressive disorder, was brought to the emergency department on 10/09/2022, two hours after ingesting approximately 500 mL of camphor oil in its liquid form. He developed two episodes of tonic-clonic seizures at home and then later had another episode in the emergency department. As he presented to the emergency room, he was confused, agitated, restless, and diaphoretic.

The management in the Emergency Department started with assessing his airway and administration of intravenous (IV) benzodiazepines and IV fluids. The ECG revealed sinus rhythm with borderline QT and QRS. During his stay in the emergency room, his mental status worsened and he became more confused and restless, and he developed another tonic-conic seizure. Therefore, he was intubated. The patient was shifted and managed in the intensive care unit, and 48 hours later the patient was extubated.

This case report illustrates the importance of addressing the potential risks of home remedies as they are increasingly being used by the population considering them as safe. Camphor, being the most cultivated essential oil, is a highly toxic compound that, even in very small concentrations, can be lethal to infants and children. It is a component of numerous over-the-counter remedies and has the potential for accidental consumption. Generalized tonic-clonic seizure being the most prominent manifestation which can occur as early as five minutes after exposure needs to be anticipated and treated accordingly. Treatment for symptomatic patients is primarily supportive with special attention paid to QRS complex widening in the ECG.

## Introduction

Camphor is a highly toxic ingredient that can be found in commonly used rubs and preparations such as Tiger balm and Vicks. The main route for toxicity is through ingestion. Onset of symptoms can occur as early as 15 minutes after ingestion ranging from sweating and agitation to seizures, cardiac arrhythmias, and cardiopulmonary arrest [1,2].

## Case presentation

We present a 61-year-old male, who is a known case of major depressive disorder, was brought to the emergency department on 10/09/2022, two hours after ingesting approximately 500 mL of camphor oil in its liquid form.

At home, 10 minutes after the ingestion he developed two episodes of tonic-clonic seizures. Each episode lasted around 2-3 minutes and aborted spontaneously. His past medical history was significant for hypertension, diabetes mellitus in addition to major depressive disorder and obsessive-compulsive disorder for which he was on sertraline, mirtazapine, clonazepam, promethazine and duloxetine, however, his compliance to medications was unknown and patient was following up in another health facility.

As he presented to the Emergency Department, he was confused, agitated, restless and diaphoretic. His Glasgow Coma Score (GCS) was 14/15. Vitals were as follows; blood pressure (BP) 133/63, heart rate (HR) 118, respiratory rate (RR) 20, oxygen saturation (SPO2) 90% at room air temp 36.6°C.

He was obeying simple commands and oriented to self and person. However, he was not comprehensive and was not vocal when asked direct questions.

A systemic examination revealed no abnormalities, clonus, or muscle rigidity.

**Figure 1:**
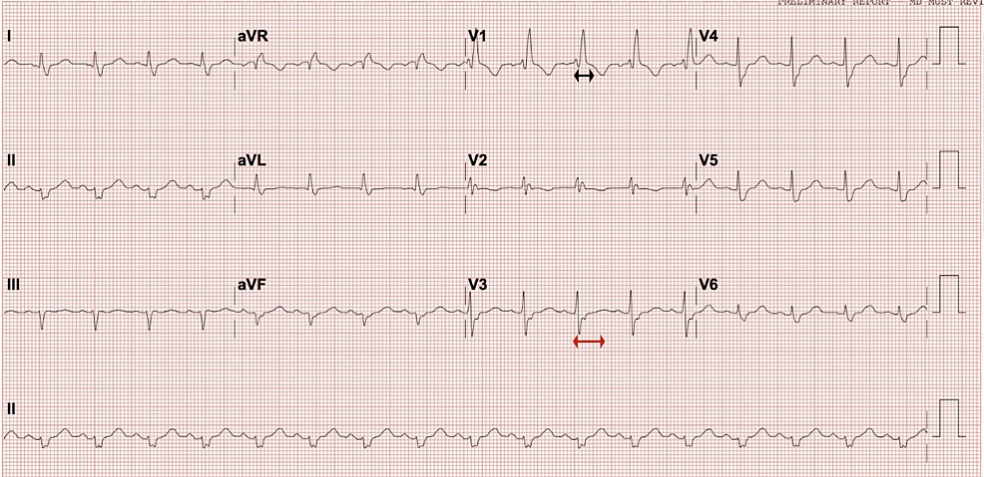
Electrocardiogram showing borderline QT prolongation and widening of the QRS. Red arrow: QTc of 503 milliseconds and black arrow: QRS of 136 milliseconds

The management in the Emergency Department, after assessing for airway patency, consisted of intravenous (IV) benzodiazepines and IV fluids. The electrocardiogram (ECG) revealed sinus rhythm with borderline QT and QRS as seen in Figure 1.

CT scan of the brain showed no evidence of intracerebral or extracerebral bleed, no cerebral parenchymal area of abnormal density and normal appearance of the ventricular system.

Initial venous blood gases showed metabolic acidosis with a very high lactate level as can be seen in Table 1.

**Table 1:** Venous blood gases PCO2: Partial pressure of carbon dioxide, PO2: Partial pressure of oxygen, HCO3: Bicarbonate International Emergency Medicine Education Project: Arterial and Venous Blood Gas Analysis. Accessed September 24, 2023: https://iem-student.org/arterial-and-venous-blood-gas-analysis/ [3]

Name	Value	Reference value
PH	7.019	7.31-7.41
PCO2	36.6 mmHg	35-45 mmHg
PO2	57.1 mmHg	30-40 mmHg
HCO3	9.1 mmol/L	22-25 mmol/L
Lactic Acid	21 mmol/L	<2.0 mmol/L
Base Excess	19.8 mmol/L	-4 to +2

During his observation in the Emergency Department, the patient became increasingly confused and restless, and he experienced another tonic-conic seizure. Therefore, the decision for intubation was made and successfully performed.

The patient was started on IV sedation and received supportive management during his stay in the intensive care unit.

Sedation was withdrawn 48 hours later, and the patient began regaining awareness; he was successfully extubated on September 12, 2022.

During his hospital stay, he was evaluated by the psychiatry and toxicology departments before being transported to another facility to continue his psychiatric treatment.

## Discussion

Essential oils have grown in popularity in recent decades, since many people believe natural goods are safer and more environmentally friendly. Camphor being the most cultivated essential oil, is a highly toxic compound that, even in very small concentrations, can be lethal to infants and children. It is a component of numerous over-the-counter remedies (Vicks Vaporub, camphor-phenol oral rinse, etc.) and has the potential for accidental consumption. Historically, it was extracted from the stem barks of the plant *Cinnamomum camphora* and used as insecticides and to preserve wood furniture [1,2,4].

This plant can be utilized to treat muscle strains, inflammation, and rheumatic disorders in the medical sector.

As we explored the literature, we discovered that the majority of cases were reported in the paediatric group as accidental ingestion because it is contained in many over-the-counter medications that are readily available at home.

Camphor's influence on the oxidation cycle is thought to be intraneuronal, occurring at the cytochrome oxidase system; however, the precise mechanism has not been determined. It is primarily a neurotoxin with a molecular structure that permits it to cross the blood-brain barrier easily. It is unknown if the toxicity of camphor is due to the original chemical, a metabolite, or both [5].

Camphor is rapidly absorbed after ingestion from the gastrointestinal tract, its half-life is thought to be approximately 167 minutes (200 mg camphor ingested alone); 93 minutes (200 mg camphor ingested with a solvent), resulting in a wide range of symptoms including but not limited to emesis, agitation, confusion, respiratory depression and most importantly generalized tonic-clonic seizure being the most prominent manifestation which can occur as early as five minutes after exposure. Patients usually present with severe nausea, vomiting, lethargy, ataxia, and convulsions. Patients who have consumed more than 30 mg/kg of a camphor-containing product or who are displaying symptoms of moderate to severe toxicity must be monitored and treated [1,5,6].

As the patient may be in an active generalized tonic-clonic seizure, management in the Emergency Department begins with securing the airway. Convulsions should be treated with a benzodiazepine. Apnea can ensue after intake; in such circumstances, early intubation to protect the airway is recommended to prevent further central nervous system injury.

There are no antidotes for camphor and enhanced elimination options are not recommended due to the high volume of distribution and high protein binding. The American Association of Poison Control Centre does not therefore recommend either activated charcoal or gastric lavage for camphor poisoning due to its rapid absorption through the gastrointestinal tract [5,7].

Treatment for symptomatic patients is primarily supportive. The cardiac monitor and ECG must be initiated to detect any potential arrhythmias, with special attention paid to QRS complex widening in the ECG. Patients should be closely monitored for the development of seizures and central nervous system (CNS) depression because it inhibits the secretion of catecholamines due to its characteristic blockage of nicotinic acetylcholine receptors, which play an important role in neurological activities, necessitating constant monitoring of the state of consciousness. Asymptomatic patients who have had topical exposure to camphor products should have their skin thoroughly cleaned with soap and water, and the patient should be monitored at home for the development of symptoms [5,7].

Parental education regarding this hazardous and potentially fatal substance found in many homes, as well as keeping it out of reach of children, is critical for preventing camphor poisoning. Camphor poisoning should be considered as a possible cause of seizures in otherwise healthy youngsters.

## Conclusions

This case demonstrates that the toxicity of essential oils and over-the-counter home remedies is a significant public health issue. It also emphasizes the significance of anticipating and being cautious about the most common complications emerging in seizures and arrhythmias in order to prevent them and abort them if they arise.
